# Effects of Metformin on CIMT and FMD in PCOS patients: a systematic review and meta-analysis

**DOI:** 10.1186/s12905-024-03275-w

**Published:** 2024-07-26

**Authors:** Fang Wang, Yici Yan, Dongying Wang, Qingnan Fan, Fangyu Yi, Xinyan Yang, Jin Lu

**Affiliations:** 1 Obstetric and Gynecologic Department, Hangzhou Women’s Hospital, Hangzhou, Zhejiang China; 2 The First Affiliated Hospital of Zhejiang Chinese Medical University (Zhejiang Provincial Hospital of Chinese Medicine), Hangzhou, Zhejiang China; 3 The First Clinical Medical College, Zhejiang Chinese Medical University, Hangzhou, Zhejiang China; 4 The Third Clinical Medical College, Zhejiang Chinese Medical University, Hangzhou, Zhejiang China; 5 Department of Nephrology, Hangzhou Hospital of Traditional Chinese Medicine, Hangzhou, Zhejiang China; 6 Obstetric and Gynecologic Department, Changxing Maternal and Child Health Hospital, No.861, Mingzhu Road, Changxing, Huzhou, 313100 Zhejiang China

**Keywords:** Metformin, Polycystic ovary syndrome, Intima media thickness, Flow-mediated dilation, Nitroglycerin-mediated dilation, Endothelial function

## Abstract

**Background:**

This study aims to analyze the efficacy of metformin on carotid intima media thickness (CIMT) and flow-mediated dilation (FMD) for patients with polycystic ovary syndrome (PCOS).

**Methods:**

A literature search of PubMed, Embase, and the Cochrane Library from inception to December 2023 was conducted. Then, after studies selection and data extraction, the mean difference (MD) with a 95% confidence interval (CI) was used to evaluate metformin efficacy in CIMT and FMD for PCOS patients. Heterogeneity was investigated through subgroup and sensitivity analysis. The protocol of our study has been registered in PROSPERO (CRD42024497239).

**Results:**

A total of 12 studies with 248 patients were included. CIMT was lower in the endpoint group (after metformin) compared with the baseline group (before metformin) (MD = -0.11, 95% CI = -0.21 to -0.01, *p* = 0.04). FMD was higher in the endpoint group compared with the baseline group (MD = 3.25, 95% CI = 1.85 to 4.66, *p* < 0.01). No statistically significant difference was observed in nitroglycerin-mediated dilation (NMD) between the two groups (MD = 0.65, *p* = 0.51). Subgroup analysis showed that a relatively lower MD of CIMT in PCOS patients from Europe in the endpoint group compared with the baseline group (MD = -0.09, 95% CI = -0.14 to -0.04, *p* < 0.001). However, the MD in CIMT was not significantly different between the endpoint group and baseline group in PCOS patients from Asia (*p* = 0.270).

**Conclusion:**

Metformin may have a beneficial effect on CIMT and FMD, but not on NMD, suggesting that metformin may help reduce cardiovascular events in PCOS patients. Notably, the clinical efficacy of metformin can be influenced by regional differences and study types.

**Supplementary Information:**

The online version contains supplementary material available at 10.1186/s12905-024-03275-w.

## Introduction

With a prevalence of 5.2%, polycystic ovary syndrome (PCOS) is a common endocrinopathy characterized by irregular menstrual periods, androgen excess, and polycystic ovaries [1]. Women with PCOS also bear a risk for the development of cardiovascular disease (CVD) and type 2 diabetes (T2D) [2]. On the one hand, from a molecular level, triglycerides (TGs), low-density lipoprotein cholesterol (LDL-C), high-density lipoprotein cholesterol (HDL-C), total cholesterol (TC), and C-reactive protein (CRP) can serve as biomarkers for predicting CVD [3, 4]. On the other hand, zooming out to a macro level, carotid intima-media thickness (CIMT) and brachial artery flow-mediated dilation (FMD) are non-invasive markers for CVD risk assessment [5]. CIMT assesses the extent of atherosclerosis or the buildup of plaque in the arteries [6]. Increased CIMT is an early indicator of atherosclerosis, which is the buildup of plaques in the arterial walls [7]. FMD measures the health and function of blood vessels, particularly the endothelium [8]. The endothelium plays a crucial role in maintaining vascular health by regulating blood flow, inflammation, and thrombosis [9]. Studies have shown that increased CIMT is associated with a higher risk of future cardiovascular events, such as heart attack and stroke [10–12]. Therefore, reducing CIMT and increasing FMD may help improve vascular health.

Metformin is one of the most crucial first-line therapeutic agents for T2D [13]. Beyond its role in diabetes treatment, the use of metformin in the treatment of PCOS is gaining growing acceptance and prevalence [14]. Recent studies show that metformin not only ameliorates insulin sensitivity but also improves the metabolic profile such as TC and LDL-C in PCOS [15–17]. This may contribute to a more balanced lipid profile and lower CVD risk. However, the cardiovascular protective effect of metformin on PCOS remains to be further demonstrated. Thereby, a meta-analysis was conducted to investigate the effect of metformin on CVD risk as assessed through CIMT and FMD in individuals with PCOS based on the published original studies.

## Methods

### Search strategy

This analysis was performed following the Preferred Reporting Items for Systematic Reviews and Meta-Analyses (PRISMA 2020) statement [18]. To examine the effect of metformin on CIMT and FMD for PCOS, two investigators conducted a comprehensive literature search in the following electronic databases from inception to December 2023: PubMed, Embase, and Cochrane. The following search terms ‘PCOS’ AND ‘Metformin’ AND (‘CIMT’ OR ‘FMD’) were employed with no restriction of countries and language. The complete search string was introduced in Supplementary Table S3. The reference lists of key articles were also scanned to identify potential studies that were not captured by the search queries. The protocol of our study has been registered in PROSPERO (CRD42024497239).

### Study selection

The inclusion criteria were as follows: (1) patients were diagnosed with PCOS based on Rotterdam criteria, National Institutes of Health (NIH), or Androgen Excess Society (AES) criteria at any age with any body mass index (BMI); (2) studies assessed the efficacy before and after metformin treatment; (3) outcomes included CIMT or FMD; (4) included studies encompassed both clinical trials and observational studies.

Meanwhile, articles were excluded if they met one of the following conditions: (1) articles were presented in abstract only, without the full original text; (2) the data were unavailable; (3) if the analysis incorporated studies with identical participant groups, preference was given to the updated study.

### Data extraction

Two independent investigators extracted data from the included articles using a standardized predesigned data extraction form. Any disagreements were resolved by discussion with a third reviewer. The relevant data were as follows: (1) study characteristics: authors, publication year, study type, country, sample size, regimens, follow-up duration, age, and BMI; (2) outcomes: CIMT and FMD are the primary outcomes and nitroglycerin-mediated dilation (NMD) is the secondary outcome.

### Quality assessment

As for RCTs, the quality assessment of clinical trials was performed by two reviewers according to the tools of the Cochrane Handbook for Systematic Reviews of Interventions. Individual quality items included selection bias (randomization sequence generation), selection bias (allocation concealment), performance bias (blinding of participants and personnel), detection bias (blinding of outcome assessment), attrition bias (incomplete outcome data), reporting bias (selective reporting), and other biases. Each bias item was defined as ‘Low Risk’, ‘High Risk’, or ‘Unclear Risk’. RevMan 5.4 was used for this section, differences of opinion were registered and resolved through consensus.

As for non-RCTs and cross-sectional studies, risk of bias in non-randomized studies of interventions (ROBINS-I) tool was used by two investigators to assessed the quality of the included studies. Each study was defined as ‘low risk’, ‘moderate risk’, ‘serious risk’, ‘critical risk’ or ‘no information’ respectively by considering the following characteristics covering bias due to confounding; bias in selection of study participants; bias in exposure measurement; bias due to misclassification of exposure during follow-up; bias due to missing data; bias in measurement of outcomes; and bias in selection of reported results.

### Statistical analysis

Statistical analyses of study outcomes were performed and pooled as forest plots by RevMan 5.4. For continuous outcomes, mean difference (MD) with 95% confidence interval (CI) was used to represent the effect size of pooled results. As for CIMT, MD < 0 indicated a potential beneficial effect of metformin for patients with PCOS, while MD > 0 implied the opposite. As for FMD and NMD, MD > 0 suggested metformin may have a beneficial effect for PCOS patients, while MD < 0 implied the opposite. Chi-square Q test and I^2^ statistic were used to assess statistical heterogeneity. I^2^ < 30% revealed low heterogeneity, 30% ≤ I^2^ ≤ 60% indicated moderate heterogeneity, and I^2^ > 60% represented high heterogeneity. Due to the clinical heterogeneity from the diversity of study type and difference in intervention, the random-effects model was used to improve statistical reliability. All reported P-values were two-sided and statistically significant when *P* < 0.05.

Subgroup analysis according to study types and regions was implemented to explore differences in how PCOS patients respond to metformin and potential impact of heterogeneity. Sensitivity analysis was performed to estimate the robustness of each study to different aspects from methodological bias.

## Results

### Study selection

**Fig. 1:**
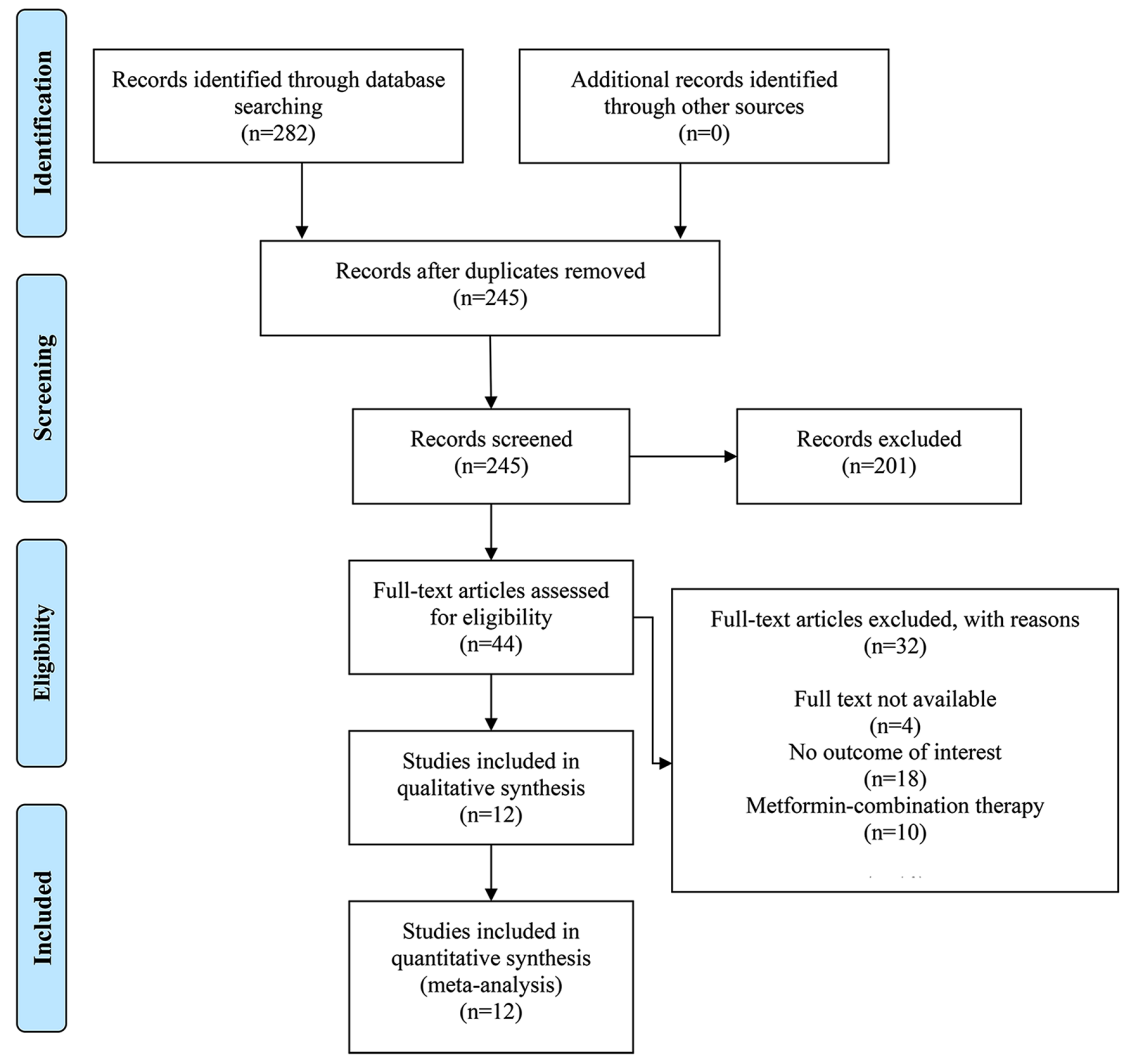
Flow diagram describing inclusion and exclusion criteria

In this study, the initial search strategy resulted in 282 citations. After eliminating duplicates, 245 articles were identified. Then, by browsing title and abstract, 44 articles were obtained. Ultimately, following a detailed examination of the full text, 32 articles were excluded. Among the exclusions, 4 studies were unavailable in full text, 18 lacked the outcome of interest, and 10 used metformin-combination therapy. A total of 12 studies [19–31] were included in the final analysis. The specific screening steps are summarized in Fig. 1.

### Study characteristics

A total of 248 patients were enrolled in our study. Five studies were randomized controlled trials (RCTs), 5 studies were non-RCTs (“non-RCT” typically refers to clinical trials lacking randomization), and the remaining 2 studies were cross-sectional studies. Metformin doses ranged from 1500 mg to 2550 mg daily. Specifically, three studies employed a dosage of 500 mg three times daily, two studies utilized 1700 mg daily, six studies opted for 850 mg twice daily, and one study implemented a daily dosage of 2550 mg. The follow-up duration for all studies, except one [21] with a 3-month follow-up, was consistently set at 6 months. The average age ranged from 21.6 to 27.0 years, and the average BMI ranged from 22.2 to 36.2 kg/m2. Three studies were from Asia, 8 studies were from Europe, and 1 study was from America. Detailed characteristics of included studies were summarized in Table 1.

**Table 1:** Characteristics of all the studies included in the meta-analysis

Author, year	Study type	Country	Follow-up duration	Intervention	No. of patients (baseline)	No. of patients (endpoint)	Age (years)	BMI (kg/m^2^)
Akram W, 2023 [19]	Cross-sectional study	Iraq	6 mo	Met (500 mg three times daily)	54	54	24.8 ± 3.5	18–30
Diamanti-Kandarakis E, 2005 [20]	Non-RCT	Greece	6 mo	Met (1700 mg daily)	20	20	25.0 ± 1.1	28.4 ± 1.6
Essah PA, 2011 [21]	RCT	United States	3 mo	Met + OC (500 mg three times daily)	11	9	26.6 ± 1.4	36.2 ± 2.5
Jensterle M, 2008 [22]	RCT	Slovenia	6 mo	Met (850 mg twice daily)	15	15	NA	29.6 ± 6.9
Kaya MG,2015 [23]	RCT	Turkey	6 mo	Met (850 mg twice daily) + Drospirenone/EE	25	25	24 ± 4	29.8 ± 6.9
Luque-Ramírez M, 2009 [24]	RCT	Spain	6 mo	Met (850 mg twice daily)	19	12	25.1 ± 6.6	30.5 ± 6.9
Naka KK, 2011 [26]	RCT	Greece	6 mo	Met (850 mg twice daily)	15	15	22.2 ± 3.6	29.4 ± 6.5
Orio Jr F, 2005 [27]	Non-RCT	Italy	6 mo	Met (850 mg twice daily)	30	30	22.8 ± 2.5	22.4 ± 2.1
Palomba S, 2010 [28]	Non-RCT	Italy	6 mo	Met (1700 mg daily) + folic acid	23	23	26.9 ± 3.1	27.9 ± 2.6
Romualdi D, 2008 [29]	Non-RCT	Italy	6 mo	Met (500 mg twice daily)	13	13	24.7 ± 4.4	22.2 ± 2.3
Sahin Y, 2007 [30]	Non-RCT	Turkey	6 mo	Met (2550 mg daily)	20	20	21.6 ± 3.7	22.4 ± 2.1
Tan BK, 2014 [31]	Cross-sectional study	Germany	6 mo	Met (850 mg twice daily)	21	21	27 (24–31)	32.8 (29.8–36.5)

RCT, Randomized Control Trail; mo, month; Met, Metformin; OC, Oral Contraceptive; EE, Ethinyl Estradiol; No, Number; NA, Not Available; BMI, Body Mass Index

### Quality assessment

According to the Cochrane Collaboration tool, all included studies were of higher quality (Supplementary Figs. S1 and S2). In terms of selection bias (random sequence generation), 5 studies were assessed as ‘low risk’. In terms of allocation concealment, 5 studies were labelled as ‘low risk’. Regarding performance bias, 3 studies were assessed as ‘low risk’ and 2 studies were deemed as ‘unclear risk’. Regarding detection bias, 3 studies were categorized as ‘low risk’ and 2 studies were deemed as ‘unclear risk’. In terms of attrition bias, 3 studies were categorized as ‘low risk’, 2 studies were assessed as ‘unclear risk’. Except for 1 study, all others were rated as ‘low risk’ for reporting bias. All 5 studies were labelled as ‘low risk’ regarding other biases.

According to the ROBINS-I tool (Supplementary Table S4), One included study was assessed as serious. Two studies were rated as moderate. The left four studies were labelled as low.

### Analysis of CIMT

**Fig. 2:**
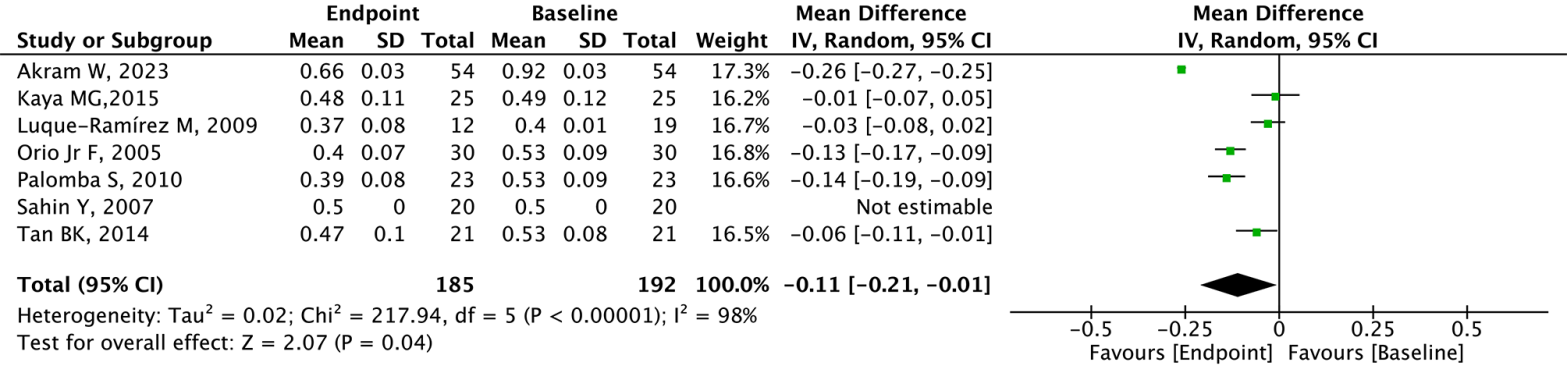
Forest plots of metformin on CIMT for PCOS patients

**Fig. 3:**
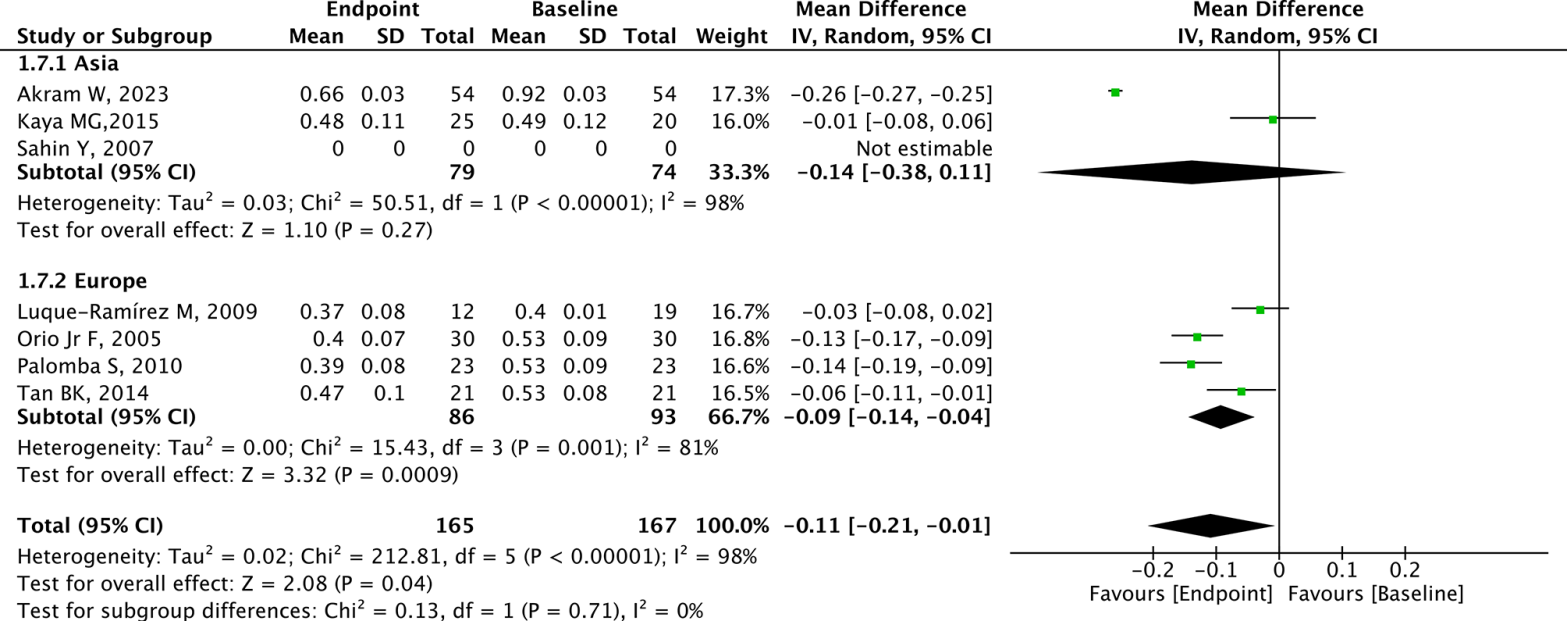
Subgroup analysis according to region on CIMT for PCOS patients

**Fig. 4:**
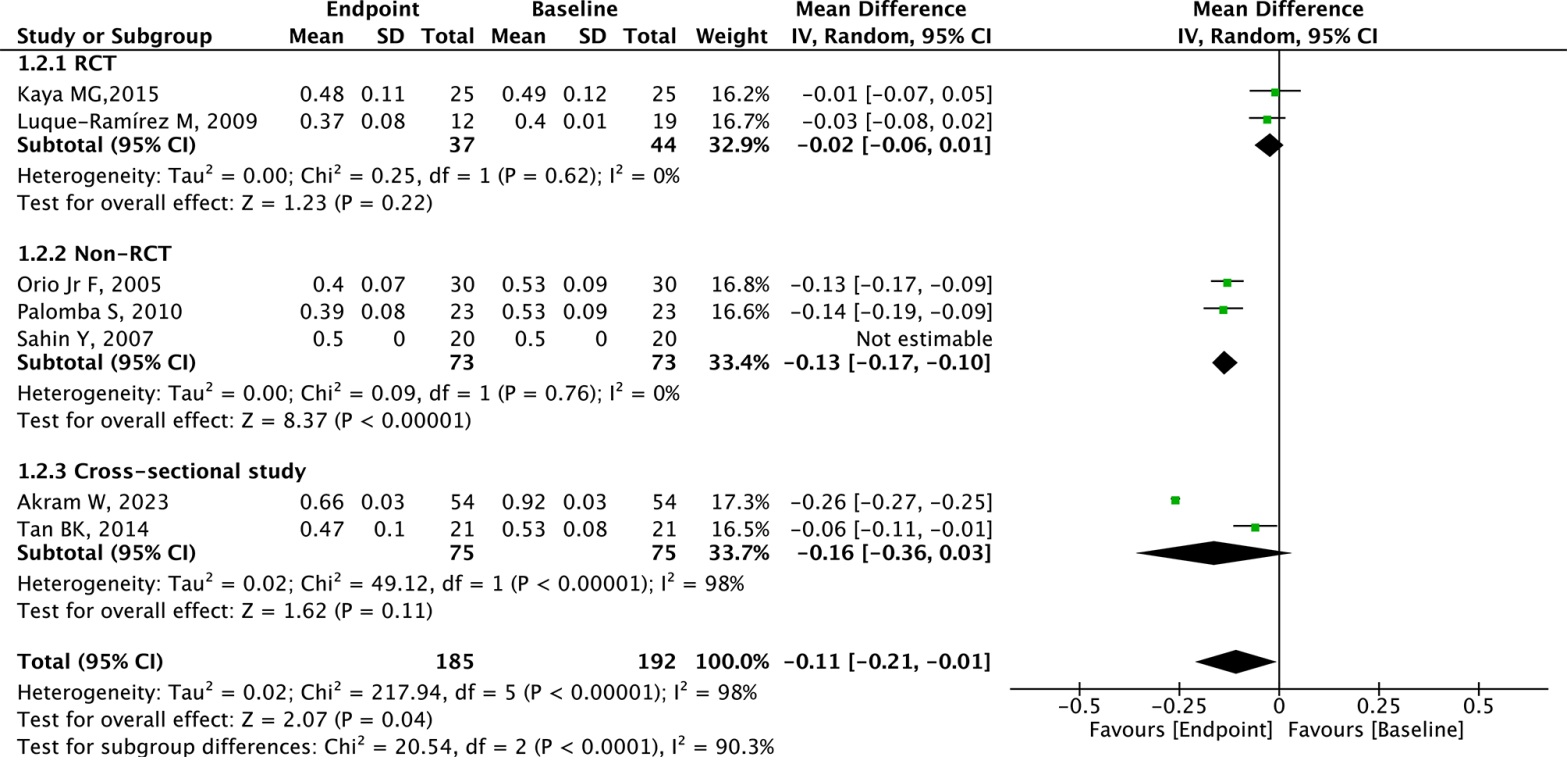
Subgroup analysis according to study type on CIMT for PCOS patients

A total of 7 studies with 192 patients provided relevant data for CIMT. CIMT was lower in the endpoint group (after metformin) compared with the baseline group (before metformin) with significant heterogeneity (MD=-0.11, 95% CI=-0.21 to -0.01, *p* = 0.04, I^2^ = 98%, Fig. 2). The summary random-effect mean difference in CIMT revealed a significant reduction among PCOS patients from Europe in the endpoint group compared to the baseline group (MD=-0.09, 95% CI=-0.14 to -0.04, *p* < 0.001, Fig. 3). However, the MD in CIMT was not significantly different between the endpoint group and baseline group in PCOS patients from Asia (*p* = 0.270, Fig. 3). Among PCOS patients from non-RCT studies, the CIMT in the endpoint group was significantly lower than that in the baseline group (MD=-0.13, 95% CI=-0.17 to -0.10, *p* < 0.001, Fig. 4). No difference in CIMT was found in RCT (*p* = 0.220, Fig. 4) and cross-sectional study (*p* = 0.110) between the two groups.

### Analysis of FMD

**Fig. 5:**
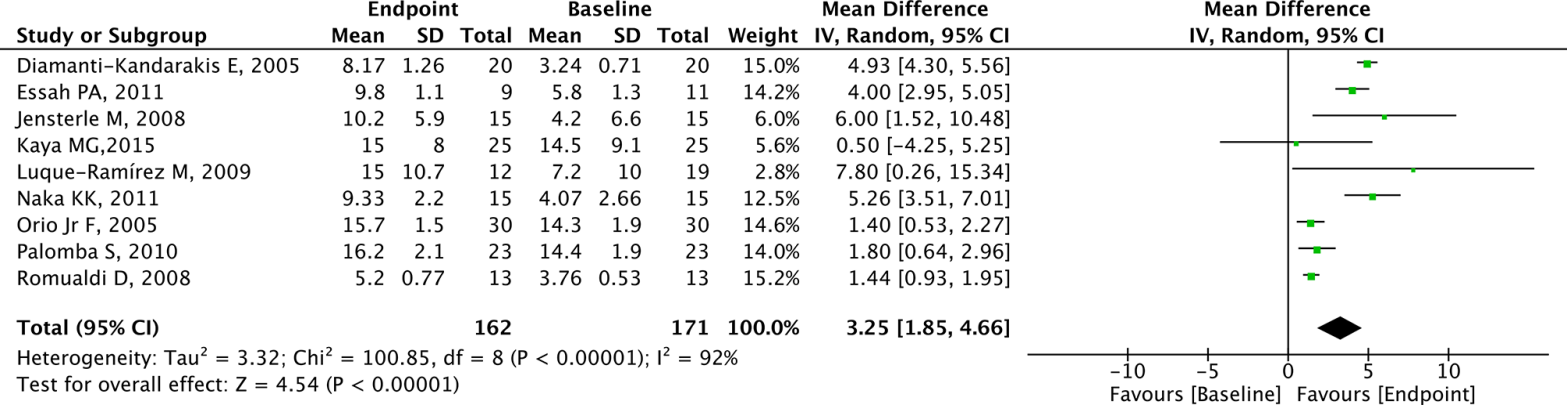
Forest plots of metformin on FMD for PCOS patients

**Fig. 6:**
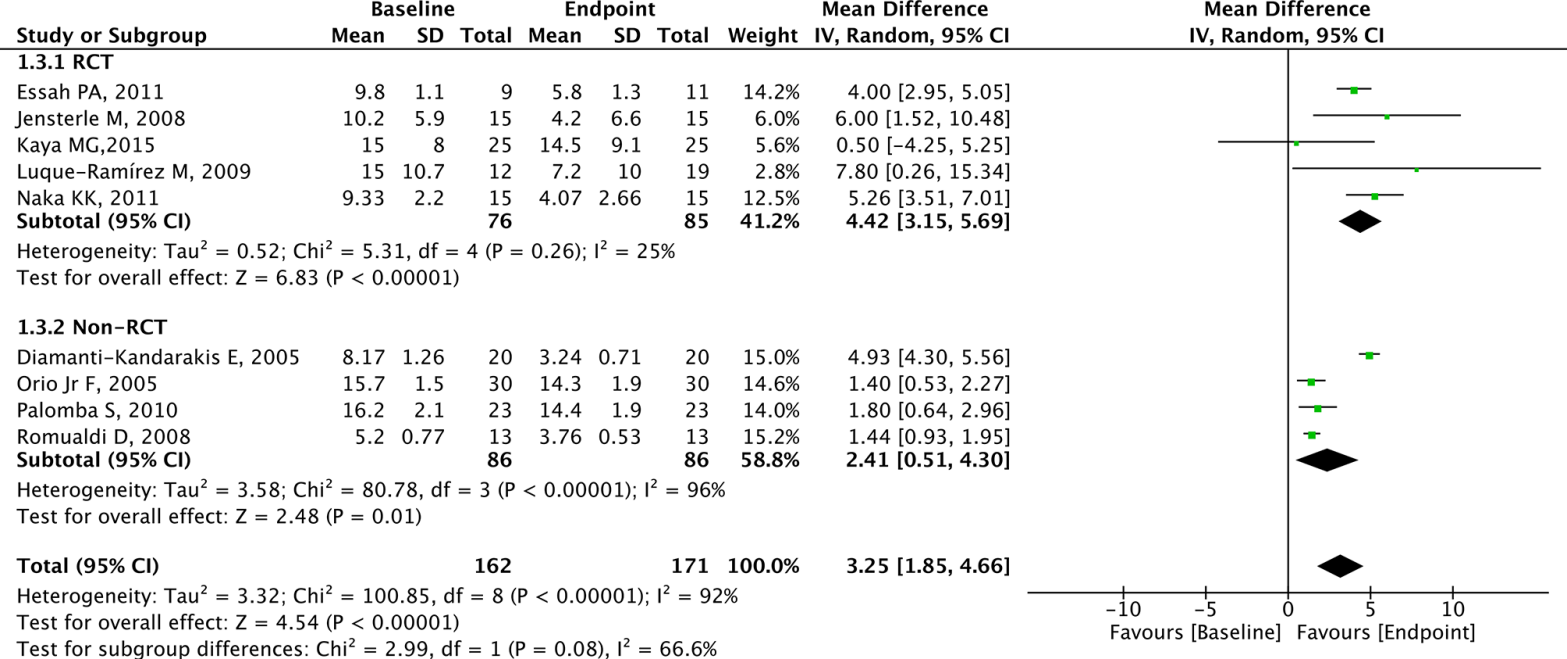
Subgroup analysis according to study type on FMD for PCOS patients

A total of 9 studies with 171 patients reported the effects of metformin on FMD. FMD was significantly higher in the endpoint group compared to the baseline group (MD = 3.25, 95% CI = 1.85 to 4.66, *p* < 0.01, I^2^ = 92%, Fig. 5). The MD in FMD revealed a significant increase among PCOS patients from the RCT group (MD = 4.42, 95% CI = 3.15 to 5.69, *p* < 0.001, Fig. 6) and non-RCT group (MD = 2.41, 95% CI = 0.51 to 4.30, *p* = 0.010, Fig. 6) in the endpoint group compared to the baseline group.

### Analysis of NMD

**Fig. 7:**
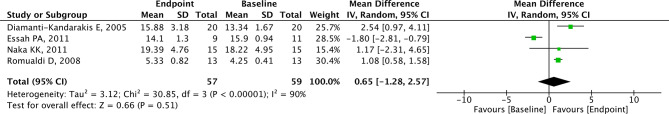
Forest plots of metformin on NMD for PCOS patients

**Fig. 8:**
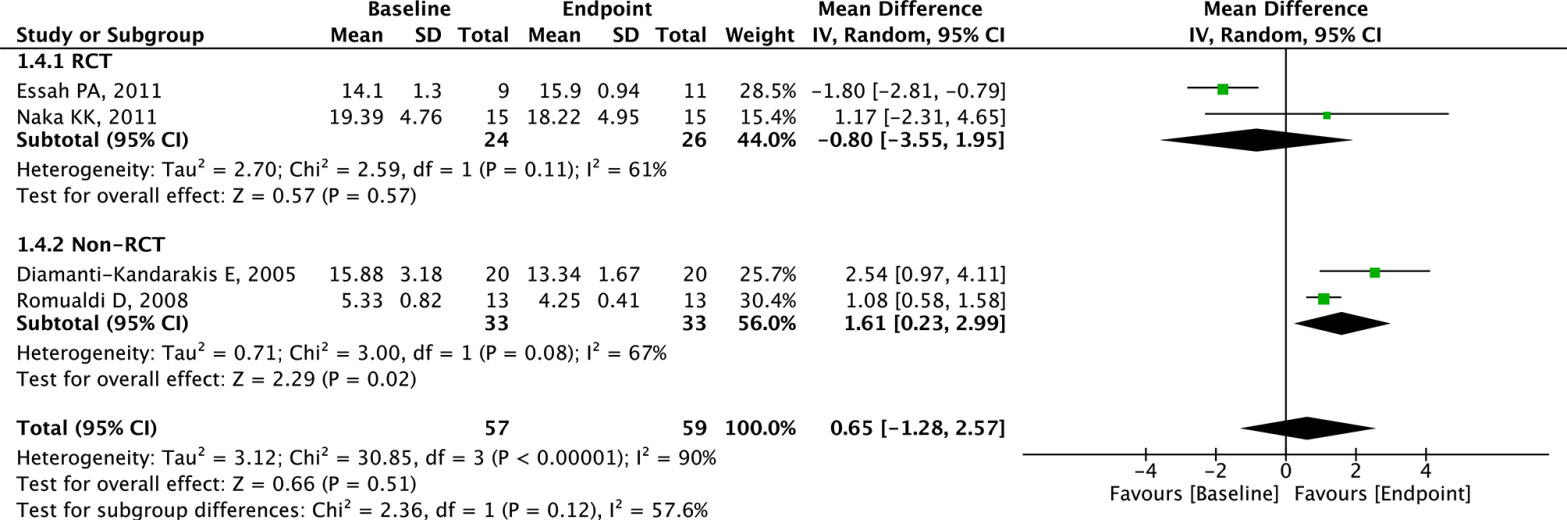
Subgroup analysis according to study type on NMD for PCOS patients

Four studies with 59 patients presented relevant data for NMD. No statistically significant difference was observed in NMD between the two groups (MD = 0.65, 95% CI=-1.28 to 2.57, *p* = 0.51, I^2^ = 90%, Fig. 7). In PCOS patients from non-RCT, NMD was higher in the endpoint group than the baseline group (MD = 1.61, 95% CI = 0.23 to 2.99, *p* = 0.020, Fig. 8). In PCOS patients from RCT, there was no statistical difference between the two groups (Fig. 8).

### Sensitivity analysis

To assess the impact of individual study outcomes on the overall results, we performed a sensitivity analysis. Through leave-one-out, the statistical results remained stable, indicating the reliability of the statistical results of this study.

## Discussion

PCOS is often associated with insulin resistance, leading to a state of hyperinsulinemia [32]. This hyperinsulinemia contributes to elevated serum testosterone, which mainly affects lipid metabolism [33]. The disturbed lipid metabolism results in acute atherosclerotic changes, increasing the risk of CVD [34]. To reduce CVD risk, metformin is a commonly used drug [35]. Metformin is an insulin sensitizer that improves glucose control and lipid metabolism [36]. Enhanced lipid metabolism, characterized by lower levels of LDL-C and TGs, and higher levels of HDL-C, contributes to a reduced risk of atherosclerosis and cardiovascular events [37]. Moreover, lowering hyperglycemia can alleviate oxidative stress and enhance nitric oxide bioavailability, contributing to improved endothelial function [38]. This improvement in endothelial function leads to better vasodilation, reduced inflammation, and decreased platelet aggregation, ultimately reducing the risk of CVD [39]. Additionally, metformin has been shown to possess anti-inflammatory properties, characterized by decreased levels of inflammatory markers such as CRP and pro-inflammatory cytokines [40]. This anti-inflammatory effect not only impedes the formation and growth of atherosclerotic plaques but also enhances endothelial function, contributing to a reduced risk of cardiovascular events [41, 42]. Therefore, the efficacy of metformin in reducing CVD risk in PCOS patients is theoretically plausible.

Previous studies have demonstrated that metformin can enhance cardiovascular health by improving lipid metabolism and inflammation at the molecular level [15–17, 43–45]. A meta-analysis conducted by Lord et al. [16] showed metformin has a significant effect in reducing LDL-C in PCOS patients. Similar results were observed in the research by Abdalla et al. [15] and Weng et al. [44], indicating that metformin had beneficial effects on LDL-C and TC, but insignificant effects on HDL-C and TG. Additionally, Chen et al. [43] and Wartena et al. [45] found decreased levels of CRP, C1q/TNF-related protein 6 (CTRP6), adiponectin, and homocysteine in patients receiving metformin. Meta-analysis by Wang et al. [44] also indicated metformin treatment could decrease CRP and interleukin 6 (IL-6) in PCOS patients. However, there is a gap in research that demonstrates, from a macro perspective, the direct improvement of cardiovascular outcomes by metformin. CIMT, FMD, and NMD are macroscopically used as indicators of CVD due to their association with endothelial dysfunction [46, 47]. CIMT is a measure of the thickness of the inner two layers of the carotid artery wall [6]. It reflects the extent of atherosclerotic plaque formation and vascular remodeling, making it a useful marker for assessing CVD risk [47]. FMD is a non-invasive measure of endothelial function. Impaired FMD indicates endothelial dysfunction, which precedes atherosclerosis and is associated with increased CVD risk [48]. By assessing endothelium-independent vasodilation, NMD complements other measures of endothelial function such as FMD and CIMT [41]. Impaired NMD suggests vascular smooth muscle dysfunction and is associated with increased CVD risk and predicts adverse cardiovascular events [49]. Therefore, our meta-analysis of 12 studies including 248 patients was performed to evaluate the efficacy of metformin on CIMT, FMD, and NMD for PCOS patients. The results demonstrated that metformin can improve both CIMT and FMD but not NMD in PCOS patients.

Metformin exerts its effects through activating AMP-activated protein kinase (AMPK), a key regulator [50]. The activation of AMPK leads to increased fatty acid oxidation and decreased hepatic gluconeogenesis, resulting in reduced hepatic lipid synthesis and secretion [51]. Moreover, AMPK activation inhibits acetyl-CoA carboxylase (ACC), a key enzyme involved in fatty acid synthesis, further reducing lipid accumulation in hepatocytes [52]. Additionally, AMPK activation by metformin promotes glucose uptake and utilization in peripheral tissues, leading to decreased circulating glucose levels and subsequent suppression of lipogenesis [53]. By suppressing lipogenesis, metformin helps prevent the development and progression of atherosclerosis, reduces the risk of plaque rupture and thrombosis, and ultimately lowers the incidence of CVD including heart attacks and strokes [54]. In terms of its anti-inflammatory properties, metformin inhibits the nuclear factor-kappa B (NF-κB) signaling pathway, reducing the production of pro-inflammatory cytokines such as tumor necrosis factor-alpha (TNF-α) and interleukin-6 (IL-6) [55]. Furthermore, by enhancing antioxidant defenses and reducing the production of reactive oxygen species (ROS), metformin reduces oxidative stress and inflammation in cardiovascular tissues, thereby lowering the risk of CVD [56].

Therefore, the American Society for Reproductive Medicine (ASRM) recommended metformin as a first-line pharmacological treatment for PCOS patients with insulin resistance, impaired glucose tolerance, or type 2 diabetes [57]. Similarly, the European Association for the Study of Diabetes (EASD) included metformin as one of the treatment options for managing metabolic abnormalities in PCOS patients, particularly those with insulin resistance or impaired glucose tolerance [58]. Consistent with the guidelines, our study found that metformin is associated with improved cardiovascular benefits in PCOS patients as evidenced by positive changes in CIMT and FMD.

The cardiovascular protection provided by metformin may vary among individuals [59]. Subgroup analysis from our study has suggested that PCOS patients from Europe were more likely to receive cardiovascular benefits when treated with metformin in terms of CIMT. In contrast, no discernible cardiovascular benefits from metformin were observed in PCOS patients from Asia. Similar results were found by a cohort study by Utzschneider et al. [60], which showed that compared with Asians, Caucasians had a higher prevalence of the monophasic glucose response curve shape when receiving metformin, indicating that metformin is more effective in controlling blood sugar in Caucasians. This variability in the effects of metformin among different races and regions could be attributed to factors such as genetic predisposition, lifestyle factors, and underlying health conditions [59, 61]. A genome-wide association study by Josephine et al. examined the association between genetic variants and metformin response across diverse populations [62]. They identified genetic factors that may contribute to variability in metformin efficacy. Since genetic differences exist among regions, Europeans and Asians may receive different benefits from metformin. Moreover, lifestyle factors can influence insulin sensitivity, glucose metabolism, and overall metabolic health, which in turn may impact the response to metformin therapy [63]. Individuals adhering to a healthy lifestyle, including a balanced diet and regular physical activity, may have a more favorable response to metformin. Conversely, those with poor lifestyle habits such as a high-fat diet, smoking, or excessive alcohol intake may experience less effectiveness from metformin treatment. Studies have shown that the Mediterranean diet is associated with numerous health benefits, including a reduced risk of heart disease, stroke, and diabetes [64]. Therefore, it is reasonable to assume that Europeans may receive more cardiovascular benefits from metformin. Furthermore, the original health conditions may differ among regions and races. A cohort study by Rabanal et al. demonstrated that women from South Asia were reported to be at a 76% greater risk of CVD than women in Norway [65]. We assume that in the context of a greater cardiovascular risk in Asia, the benefits of metformin may be overshadowed.

As for study types, subgroup analysis showed that in terms of FMD, PCOS patients from non-RCTs and RCTs receive cardiovascular benefits when treated with metformin. In terms of CIMT, PCOS patients from non-RCTs were more likely to receive cardiovascular benefits when treated with metformin. In contrast, no discernible cardiovascular benefits from metformin were observed in PCOS patients from RCTs and cross-sectional studies. In terms of NMD, PCOS patients from non-RCTs receive cardiovascular benefits when treated with metformin, but no discernible cardiovascular benefits from metformin were observed in PCOS patients from RCTs. Due to the limited number of participants in each subgroup according to study type, the results had a certain degree of contingency.

Heterogeneity posed a major challenge. Study types were considered one of the major sources of the heterogeneity. In terms of CIMT, cross-sectional studies were considered a major source of heterogeneity. Due to poor baseline comparability, cross-sectional studies are less suited for making strong causal inferences [66]. Thus, the evidence level of cross-sectional studies is generally considered to be lower when compared with RCTs and non-RCTs. In terms of FMD, non-RCTs were considered a major source of heterogeneity. Because of a lack of randomization, control for bias, and challenges in establishing causation, the evidence level of non-RCTs is relatively low compared with RCTs. Additionally, differences in study populations such as sex, BMI, age, and medication compliance may be considered as potential sources of heterogeneity. For example, in addition to taking metformin as prescribed, some individuals may also incorporate lifestyle modifications such as regular exercise and dietary adjustments to achieve better outcomes, while some may not. However, due to a lack of relevant data, this assumption remained to be proved.

To our knowledge, this is the first meta-analysis to explore the efficacy of metformin on CIMT and FMD for PCOS patients. However, there were several limitations, most of which are inherent to the meta-analysis. Firstly, the limited number of participants inevitably leads to a decrease in the credibility of the results. Secondly, the presence of moderate to high heterogeneity has somewhat reduced the robustness of our analysis. Therefore, a random-effects model was used. Thirdly, the refinement of subgroup analyses grouping was constrained by a lack of available data. Additionally, our evidence comes from single-arm studies, which limits our ability to establish causality and control for potential confounding factors.

## Conclusion

Metformin may help decrease CIMT and improve FMD, which are indicators of vascular function, suggesting potential cardiovascular benefits for PCOS patients. In RCTs, metformin may help increase FMD, but no beneficial effect of metformin on CIMT or NMD was observed in PCOS patients. In non-RCTs, metformin may help decrease CIMT and increase FMD and NMD, indicating that metformin may reduce CVD risk for PCOS patients. In cross-sectional studies, no beneficial effect of metformin on CIMT was observed. While metformin may have a beneficial effect on CIMT in PCOS patients from Europe, its efficacy in Asian patients remains to be further proved.

### Electronic supplementary material

Below is the link to the electronic supplementary material.

Supplementary Material 1

Supplementary Material 2

Supplementary Material 3

Supplementary Material 4

Supplementary Material 5

## Data Availability

The datasets supporting the conclusions of this article are included within the article.

## Abbreviations

CIMT: Carotid Intima Media Thickness
FMD: Flow-Mediated Dilation
PCOS: Polycystic Ovary Syndrome
MD: Mean Difference
CI: Confidence Interval
NMD: Nitroglycerin-Mediated Dilation
CVD: Cardiovascular Disease
T2D: Type 2 Diabetes
TGs: Triglycerides
LDL-C: Low-Density Lipoprotein cholesterol
HDL-C: High-Density Lipoprotein cholesterol
TC: Total Cholesterol
CRP: C-Reactive Protein
NIH: National Institutes of Health
AES: Androgen Excess Society
BMI: Body Mass Index
RCTs: Randomized Controlled Trials
CTRP6: C1q/TNF-Related Protein 6
IL-6: Interleukin 6
AMPK: AMP-Activated Protein Kinase
ACC: Acetyl-CoA Carboxylase
NF-κB: Nuclear Factor-kappa B
TNF-α: Tumor Necrosis Factor-alpha
ROS: Reactive Oxygen Species
ASRM: American Society for Reproductive Medicine
EASD: European Association for the Study of Diabetes
