# Supplementary figures and images for: Effects of Metformin on CIMT and FMD in PCOS patients: a systematic review and meta-analysis
Source: BMC Womens Health. 2024 Jul 26;24:426. doi: 10.1186/s12905-024-03275-w (PMC11282760; doi:10.1186/s12905-024-03275-w)

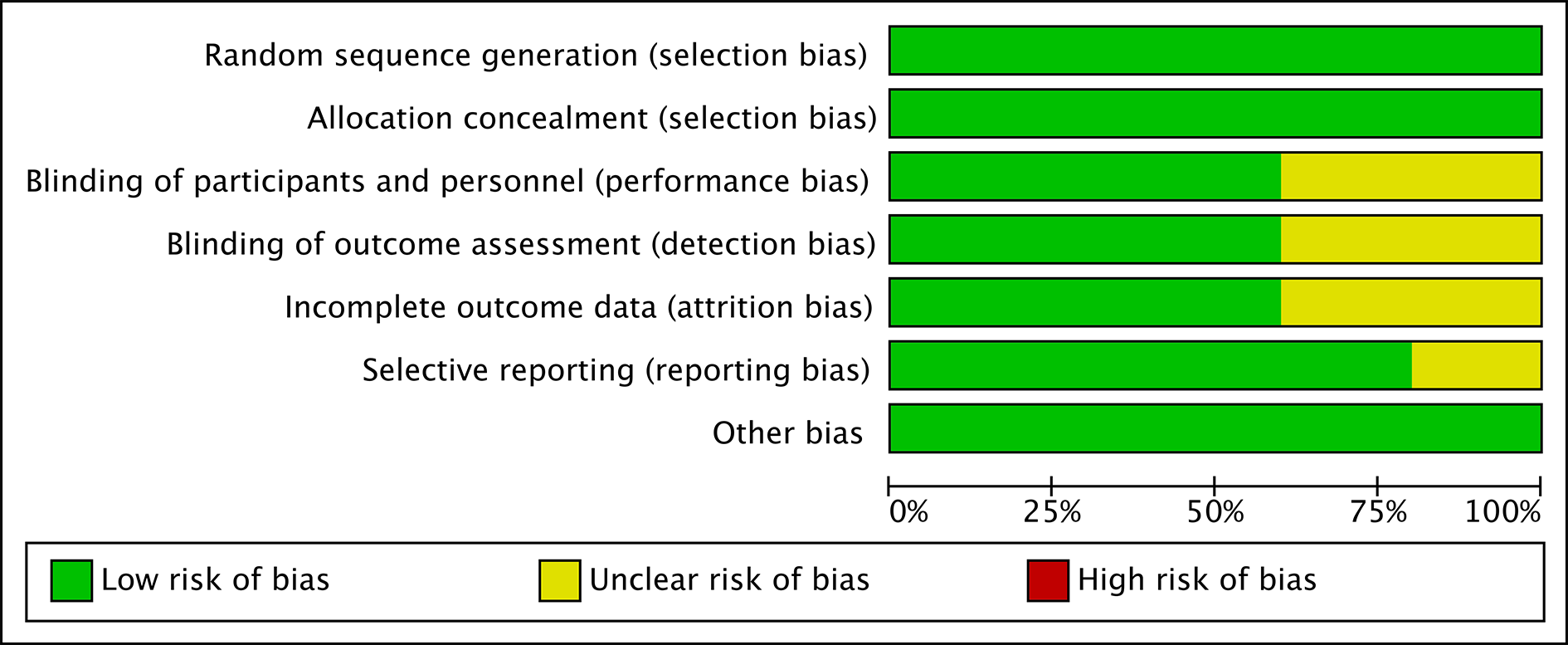

Supplement: Supplementary file 1 — Supplementary Material 1 [file 12905_2024_3275_MOESM1_ESM.png]

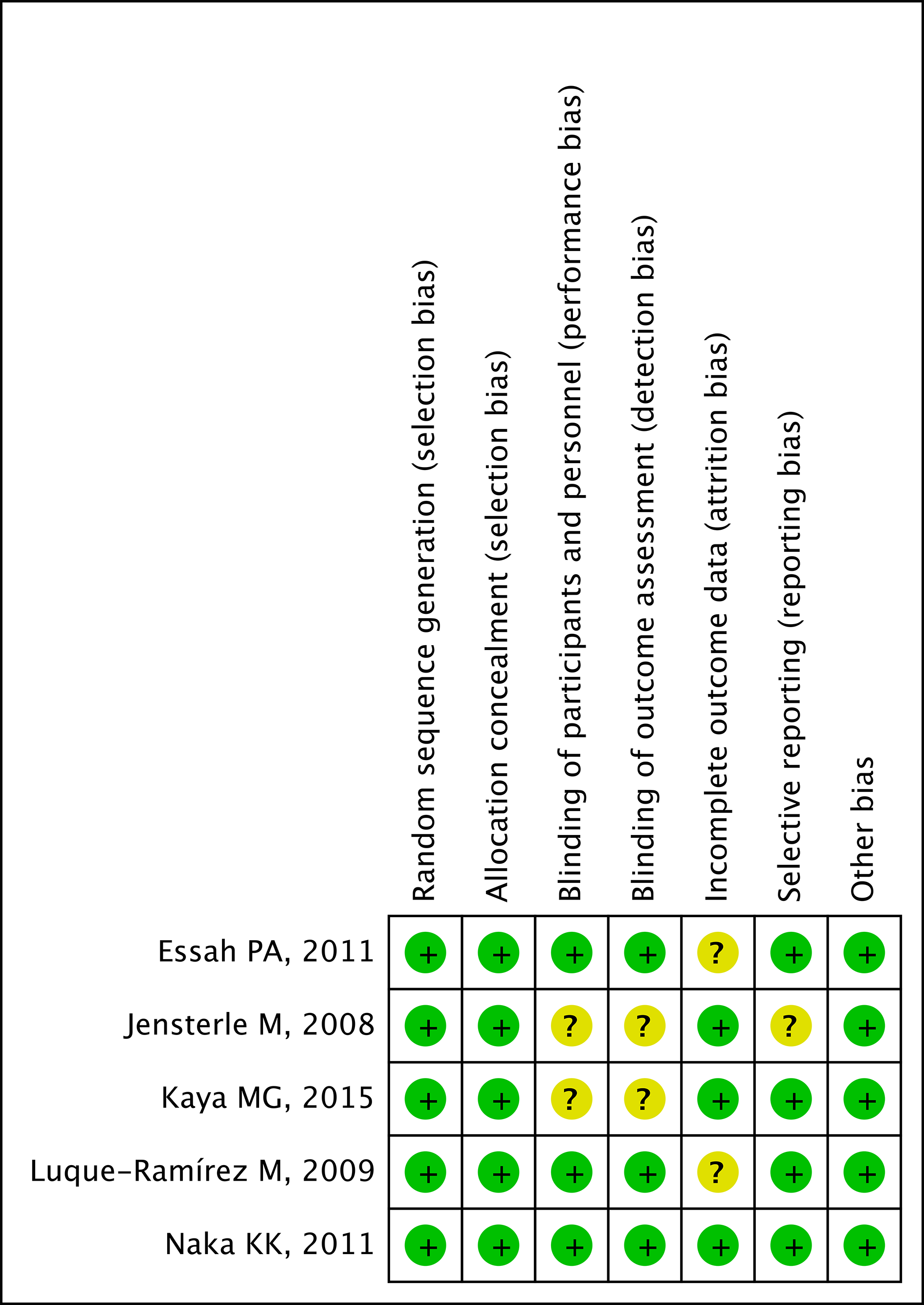

Supplement: Supplementary file 2 — Supplementary Material 2 [file 12905_2024_3275_MOESM2_ESM.png]
