# Supplementary material for: Effects of Metformin on CIMT and FMD in PCOS patients: a systematic review and meta-analysis
Source: BMC Womens Health. 2024 Jul 26;24:426. doi: 10.1186/s12905-024-03275-w (PMC11282760; doi:10.1186/s12905-024-03275-w)
Supplement: Supplementary file 3 — Supplementary Material 3 [file 12905_2024_3275_MOESM3_ESM.docx]

**Supplementary Table 1. Search Strategy.**

**Data searched from PubMed**

| Search Query | Results |
| --- | --- |
| #1 "Polycystic Ovary Syndrome"[Mesh] OR Stein-Leventhal Syndrome OR Sclerocystic Ovarian Degeneration OR Sclerocystic Ovary Syndrome OR Polycystic Ovarian Syndrome OR Sclerocystic Ovaries OR Sclerocystic Ovary | 24,198 |
| #2 "Metformin"[Mesh] OR Dimethylbiguanidine OR Dimethylguanylguanidine OR Glucophage | 30,647 |
| #3 "Carotid Intima-Media Thickness"[Mesh] OR Carotid Intima Media Thickness OR endothelial OR Flow-mediated dilation OR Flow mediated dilation | 456,795 |
| #4 #1 AND #2 AND #3 | 73 |

Searched before December, 2023. Results: 73.

**Data searched from** **Embase**

| Search Query | Results |
| --- | --- |
| #1 'ovary polycystic disease'/exp OR OR 'stein-leventhal syndrome' OR ('stein leventhal' AND ('syndrome'/exp OR syndrome)) OR (sclerocystic AND ovarian AND degeneration) OR (sclerocystic AND ovary AND syndrome) OR (polycystic AND ovarian AND syndrome) OR (sclerocystic AND ovaries) OR (sclerocystic AND ovary) | 39,956 |
| #2 'metformin'/exp OR dimethylbiguanidine OR dimethylguanylguanidine OR glucophage | 92,734 |
| #3 'carotid intima-media thickness'/exp OR 'carotid intima media thickness' OR Flow mediated dilation OR endothelial | 552,181 |
| #4 #1 AND #2 AND #3 | 182 |

Searched before December, 2023. Results: 182.

**Data searched from Cochrane Library**

| Search Query | Results |
| --- | --- |
| #1 MeSH descriptor: [Polycystic Ovary Syndrome] explode all trees | 1,933 |
| #2 (Stein-Leventhal Syndrome):ti,ab,kw OR (Sclerocystic Ovarian Degeneration):ti,ab,kw OR (Sclerocystic Ovary Syndrome):ti,ab,kw OR (Polycystic Ovarian Syndrome):ti,ab,kw OR (Sclerocystic Ovaries):ti,ab,kw OR (Sclerocystic Ovary):ti,ab,kw | 2,222 |
| #3 MeSH descriptor: [Metformin] explode all trees | 5,011 |
| #4 (Dimethylbiguanidine):ti,ab,kw OR (Dimethylguanylguanidine):ti,ab,kw OR (Glucophage):ti,ab,kw | 252 |
| #5 MeSH descriptor: [Carotid Intima-Media Thickness] explode all trees | 392 |
| #6 (Carotid Intima Media Thickness):ti,ab,kw OR (endothelial):ti,ab,kw OR (Flow mediated dilation) :ti,ab,kw | 22,585 |
| #7 #1 OR #2 | 3,473 |
| #8 #3 OR #4 | 5,166 |
| #9 #5 OR #6 | 22,585 |
| #10 #7 AND #8 AND #9 | 27 |

Searched before December, 2023. Results: 27.
