# Supplementary material for: Effects of Metformin on CIMT and FMD in PCOS patients: a systematic review and meta-analysis
Source: BMC Womens Health. 2024 Jul 26;24:426. doi: 10.1186/s12905-024-03275-w (PMC11282760; doi:10.1186/s12905-024-03275-w)
Supplement: Supplementary file 4 — Supplementary Material 4 [file 12905_2024_3275_MOESM4_ESM.docx]

**Supplementary Table 2. Quality assessment of the included publications, based on the ROBINS-I tool (low, moderate, serious, critical, no information).**

| **Author, year** | **Types of bias** | | | | | | | **Overall rating** |
| --- | --- | --- | --- | --- | --- | --- | --- | --- |
|  | **Bias due to confounding** | **Bias due to selection of participants** | **Bias due to exposure assessment** | **Bias**  **due to**  **misclassification**  **during follow-up** | **Bias due to missing data** | **Bias**  **due to measurement of the outcome** | **Bias**  **due to**  **selective reporting**  **of the**  **results** |  |
| Akram W, 2023 | Serious | Moderate | Moderate | Low | Low | Low | Low | Serious |
| Diamanti-Kandarakis E, 2005 | No information | Low | Low | Low | Low | Low | Low | Low |
| Orio Jr F, 2005 | Moderate | Low | Low | Low | Low | Low | Low | Low |
| Palomba S, 2010 | Low | Low | Low | Low | Low | Low | Low | Low |
| Romualdi D, 2008 | No information | Low | Low | Low | Low | Low | Low | Low |
| Sahin Y, 2007 | Low | Moderate | Low | Low | Low | Moderate | Low | Moderate |
| Tan BK, 2014 | Low | Moderate | Moderate | Low | Low | Low | Low | Moderate |
